# Research interrupted: The impact of the COVID-19 pandemic on multiple sclerosis research in the field of rehabilitation and quality of life

**DOI:** 10.1177/20552173211038030

**Published:** 2021-08-26

**Authors:** Rebecca Maguire, Sinead Hynes, Barbara Seebacher, Valerie J Block, Kathy M Zackowski, Johanna Jonsdottir, Marcia Finlayson, Prue Plummer, Jennifer Freeman, Barbara Giesser, Gloria von Geldern, Michelle Ploughman

**Affiliations:** Department of Psychology, Maynooth University, County Kildare, Ireland; School of Health Sciences, National University of Ireland Galway, Galway, Ireland; Clinical Department of Neurology, Medical University of Innsbruck, Innsbruck, Austria; Department of Neurology, University of California, San Francisco, CA, USA; Weill Institute for Neurosciences, San Francisco, CA, USA; Patient Management Care and Rehabilitation Research, National Multiple Sclerosis Society, New York, USA; IRCCS Fondazione Don Carlo Gnocchi, Milan, Italy; School of Rehabilitation Therapy, Queen’s University, Kingston, ON, Canada; Department of Physical Therapy, MGH Institute of Health Professions, MGH Institute of Health Professions, Boston, MA, USA; School of Health Professions, University of Plymouth, Plymouth, UK; Staff Physician, Pacific Neuroscience Institute, Santa Monica, CA, USA; Department of Neurology, University of Washington, Seattle, WA, USA; Faculty of Medicine, Memorial University of Newfoundland, Labrador, Canada

**Keywords:** Multiple sclerosis, quality of life, rehabilitation, barriers to research, gender, COVID-19

## Abstract

**Background:**

The COVID-19 pandemic has likely had a negative impact on rehabilitation and quality of life (QoL) research in multiple sclerosis (MS).

**Method:**

We explored perceived barriers to research among 87 researchers, representing 18 countries, both prior to and since COVID-19.

**Results:**

A Wilcoxon signed-rank test found that significantly more researchers reported experiencing barriers to research since the onset of the pandemic compared to pre-COVID-19 (p < .001), with 78% of respondents reporting at least some barriers since COVID-19. The most commonly-cited barriers related to participant access (n = 38) and interruptions/delays to projects (n = 19). Although no gender differences were found in the number of barriers reported, female respondents were more likely to cite time or competing demands as barriers to research. Females were also more likely to perceive being negatively impacted by the pandemic compared to other genders (p = .007).

**Conclusions:**

Implications for the future landscape of rehabilitation research in MS are discussed.

## Introduction

The COVID-19 pandemic has impacted almost all researchers,^1^ with more detrimental effects on some research programmes compared to others. While much work has established how the management of multiple sclerosis (MS) has changed in the last year,^2^ with a shift towards virtual consultations,^3,4^ the impacts of COVID-19 on MS research are less understood. In the context of MS, research in the field of rehabilitation and quality of life (QoL) is likely to have been particularly affected given the high reliance on face-to-face data collection and intervention delivery.

In this study, we aimed to explore the barriers encountered by researchers working in rehabilitation and QoL research in MS, both prior to and since the COVID-19 pandemic. We further explored whether barriers varied by gender, given the known disadvantage of female researchers in other domains.^5,6^ Understanding the challenges faced by researchers is important when anticipating the future of rehabilitation research and interventions designed to support people with MS (PwMS).

## Method

### Sample

MS QoL and/or rehabilitation researchers were invited to participate in the study in January-February 2021 via professional bodies and through networks of members of the International Women in MS rehabilitation (IWiMS) group, using a mixture of purposive and snowball sampling. Specifically, invitations were sent to professional contacts of the study authors and to other members of the rehabilitation subgroup of IWiMS. Examples of groups contacted were: members of the euRIMS (Rehabilitation in MS) special interest group in mobility, the euRIMS special interest group in occupation, the All-Ireland MS Research Network, rehabilitation researchers who attended the MS Society of Canada 2019 conference, members of various MS research groups in Austria, Belgium, Finland, Turkey, the UK, Switzerland and Germany, among others, and a wide range of rehabilitation researchers, physiotherapists, physicians, and other allied health professional researchers known to the authors. While invitations were mostly made via email, additional vocal invitations were made during remote meetings of certain research groups. Further calls for participants were made using Twitter, with tweets shared among the followers of relevant networks and MS societies. In order to be eligible to participate, respondents had to have conducted research in the area of MS rehabilitation or QoL. No restrictions were placed on career stage or nature of employment. Ethical approval for the study was granted from Maynooth University in December 2020 (SRESC-2020-2422005).

### Measures

A cross-sectional online survey was developed to collect quantitative and qualitative data from researchers using Qualtrics, v.2021 (Qualtrics, Provo, UT). This included the collation of sociodemographic information such as gender (male, female, non-binary, other), country of residence (open text response) and employment status (full-time or part-time, and contract type). In addition, information on career stage was obtained, with respondents asked to indicate if they were (1)^[Bibr bibr1-20552173211038030]^still in training, (2) an early-career researcher (5 years or less since their first research/academic appointment), (3) a mid-career researcher (6-12 years since first appointment), (4) a senior researcher (>12 years since first appointment), or (5) other. Respondents also indicated the number of years’ experience they had in MS research using an open-text response, as well as the percentage of their working time spent of research activities each week. Separately, respondents were asked to indicate if they had any caring responsibilities.

In relation to their experience of barriers to research, researchers were asked to report on the extent of barriers encountered both prior to and since COVID-19. Specifically, they were asked “Before COVID-19, did you encounter any barriers in conducting research in rehabilitation/QOL in MS?”, with four possible options: “Yes, to a great extent”, “Yes, to some extent”, “No”, and “Unsure”, with the latter two categories combined for the purpose of analysis, such that scores ranged from 1–3, with higher scores denoting a greater experience of barriers. An open-text question requested more detail on barriers from respondents using the following phrasing: “If yes, outline the main barriers to conducting research before COVID-19”. The same two questions were then asked again, but in relation to experience of barriers since COVID-19.

In order to establish perceptions of gender advantage/disadvantage in light of COVID-19, respondents were asked to rate their agreement with the following statement “When compared to researchers of other genders, I have experienced greater difficulties in conducting research in MS rehabilitation/QoL following COVID-19”, with responses ranging from 1 (strongly disagree) to 5 (strongly agree). Further details on all the above questions are included in the supplementary appendix.

### Analysis

Descriptive statistics were calculated for sociodemographic/employment information and extent of barriers encountered prior to and since COVID-19. Given the non-normal distribution of the data, a Wilcoxon signed-ranks test was used to compare the extent of the barriers experienced by researchers prior to and since COVID-19. Barriers described in response to the open-text questions were categorised independently by two coders (RM, SH), with a high level of agreement (88%); any discrepancies were resolved with input from a third coder (BS). Where respondents mentioned more than one barrier, these were separately coded. Following categorisation into broad themes, additional subthemes were identified (Table 1). Independent t-tests were used to compare gender differences in the number of barriers mentioned both prior to and since COVID-19, with a further independent-test test used to compare male and female perceptions of gender advantage or disadvantage in light of COVID-19.

**Table 1.** Barriers encountered by researchers both before and since COVID-19.

**Barriers before COVID-19**		
**Superordinate Barrier pre-COVID-19**	**Subordinate Barrier pre-COVID-19**	**Representative Quotes**
Funding (n = 19; male = 4, female = 15)	Difficulty obtaining funding	“It is getting increasingly difficult to get third party funded money for research in MS.” “Funders say they are interested in QoL, but still tend to fund basic science as a priority.” “Less sponsors compared to other fields, sometimes the H index is required. It is somewhat unfair.”
Time/competing responsibilities (n = 15; male = 1, female = 14)	Balancing commitments	“Difficult to balance research obligations with other commitments.”
	Teaching	“I work in an education heavy institution with little time allowed for research.” “Competing obligations such as teaching, curriculum development, board services, etc.”
	Clinical work	“I work in a clinical specialist role so the majority of my time is clinical. Only small amount of time for research projects.”
	Caring responsibilities	“Many positions are full time and with my caring role couldn't commit to full time.” “The travel time commitment to scientific meetings was a problem, as I need to have child care organized before any trip.”
Lack of support/opportunity (n = 15; male = 2, female = 13)	Institutional or administrative barriers	“Logistical barriers in terms of recruiting students and staff. Equipment and space limitations.” “Bureaucracy and institutional inertia”.
	Lack of collaborative opportunities	“Collaboration opportunities, and low interest in research groups in this area.”
	Difficulties publishing	“Many disease specific journals do not consider rehabilitation studies high enough impact to publish.”
	Gender or career bias	“Hard to gain independence, to be taken “seriously” by the established male old guard in the field.” “Male doctors in high positions preferred to work with male researchers.” “…a sense of fear among early and mid-career investigators of senior investigators taking credit for work/ideas.”
Participant access (n = 8; male = 3, female = 5)	General problems with recruitment	“Difficulties with subject recruitment and retention” “Access to MS population in rural communities.”
	Competition for participants	“There are many researchers focused on MS at my institution, so there is a great deal of competition for study participants.”
**Barriers since COVID-19**		
**Superordinate Barrier since COVID-19**	**Subordinate Barrier since COVID-19**	**Representative quotes**
Participant access (n = 38; male = 9, female = 29)	Restrictions on recruitment	“Significant restrictions on human subjects research.” “Poor accessibility to patients.” “COVID related restrictions mean that I cannot bring in patients to the lab for assessments, all ongoing studies have either been modified or put on hold.”
	Fear of COVID	“increased difficulty recruiting patients for studies that require in-person visits due to fear of contracting COVID.”
Barriers since COVID-19		
Superordinate barrier since COVID	Subordinate barrier	Representative quotes
Interruptions or delays (n = 19; male = 7, female = 12)	Projects interrupted	“Projects were disrupted in the middle of delivery and this will impact overall results.” “My research requires close proximity. As such, my main project is shut down for the foreseeable future.”
	Closure of facilities	“Difficulties to do testing in the center, or to have permission to conduct tests at our university.” “My lab was shut down for 4 months and is now in restricted capacity.”
	Institutional or administrative barriers	“Slower response time from ethic committee when not a specific COVID related project” “…non-COVID-19 research is being sidelined and there have been longer processing and review times.”
Time/competing demands (n = 11; male = 0, female = 11)	Caring responsibilities	“Even more difficult to dedicate sufficient time to research, especially given additional responsibilities with caring/home-schooling.” “Home schooling make it more challenging to complete work in usual hours therefore I work extra in my own time to get everything completed.” “Spending more time facilitating at home learning and providing childcare while schools were closed also significantly impacted my research.”
	Other responsibilities	“Too many administrative and clinical responsibilities which interfere with research time”
Funding (n = 8; male = 1, female = 7)	Cancellation of funding	“The usual funding competitions in my field were cancelled.” “Charitable body funding no longer currently available.”
Additional COVID-19 challenges (n = 7; male = 1, female = 6)	Working remotely	“inability to get the research team together - there are limits to virtual collaboration for some activities.” “Hard to maintain focus and motivation working full time from home.” “..having research assistants has not been helpful since COVID-19 as many tasks are not able to be completed virtually with confidentiality of research data so they are not able to help very much with many of the important tasks.”

## Results

### Sample characteristics

Of the 87 respondents, the majority were female (66, 76%), working full-time (74, 85%), at mid-late career stage (53, 61%), and had an average of 10 years’ experience in MS rehabilitation/QoL research (SD = 7.3, range 1–30 years). Respondents came from 18 different countries, including the USA (n = 24), Italy (n = 16), Canada (n = 10), Austria (n = 9), France (n = 4), the UK (n = 3), Turkey (n = 3), Ireland (n = 3), Belgium (n = 3), Norway (n = 2), Switzerland (n = 1), Spain (n = 1), Slovenia (n = 1), Israel (n = 1), Germany (n = 1), Finland (n = 1), the Czech Republic (n = 1), and Australia (n = 1). On average, respondents spent 57% (SD = 25.8) of their working week engaged in research. Most (50, 59%) reported some caring responsibilities.

### Barriers to research

**Figure 1.**
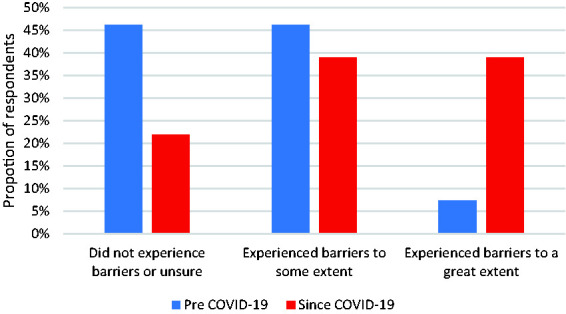
The extent to which respondents experienced barriers to research prior to and since COVID-19.^a^ ^a^Barriers more likely to be experienced since COVID-19 compared to pre COVID-19, using Wilcoxon signed-rank test (T=1314, z = –5.372, p < .001).

A Wilcoxon signed-rank test found that respondents were significantly more likely to report experiencing barriers since COVID-19 (M = 2.18) than pre-COVID-19 (M = 1.61; T = 1314, z = −5.372, p < .001), with only 22% (n = 18) reporting no barriers since COVID-19, while 39% (n = 32) reported barriers “to some extent” and a further 39% (n = 32) experiencing barriers “to a great extent”. This compares to only 6% (n = 7) who reported barriers “to a great extent” prior to COVID-19 (see Figure 1).

Of respondents who chose to complete an open text response, significantly more barriers were mentioned since COVID-19 (total n mentioned = 83) than pre-COVID-19 (total n mentioned = 57; T = 808, z = −2.765, p = .006). Independent t-tests found no gender differences in the number of barriers mentioned at either time point (p > .05), however females (M = 3.11, SD = 1.13) were significantly more likely than males (M = 2.32, SD = .95) to perceive themselves as experiencing difficulties conducting research in MS rehabilitation/QoL following COVID-19 when compared to other genders (t(79) = −2.782; p = .007).

Analysis of the open-text response revealed that, prior to COVID-19, the most commonly mentioned barriers were difficulty obtaining funding (n = 19), managing time/competing demands (n = 15), and lack of support/opportunity (n = 15). Since COVID-19, the most common barriers encountered related to participant access (n = 38), and other COVID-19 interruptions/delays to research projects (n = 18). Time/competing demands was mentioned by 11 respondents, all female, including some mentions of childcare and home-schooling responsibilities (see Table 1 for representative quotes).

## Discussion

Our analysis clearly highlights the negative impact that the COVID-19 pandemic has had on MS rehabilitation/QoL research. Unsurprisingly, limited access to participants was common, with numerous reports of trials being halted or delayed. Barriers to participant access were found to stem from logistical issues (e.g., closure of facilities), or participant characteristics themselves (e.g., fear of contracting COVID-19). Given that anxiety in PwMS has increased following the pandemic,^7,8^ difficulty accessing participants may persist for some time, however it is yet to be established whether this may be mitigated by the vaccine roll out. Furthermore, as much research in this field had to pivot to online delivery, it cannot yet be established whether this may have had any impacts on enhancing QoL in PwMS. Given the success of telemedicine and virtual consultations in the clinical management of MS,^3,4^ there is a clear need to further evaluate the efficacy of online rehabilitation interventions, which may offer a promising alternatives to face-to-face intervention delivery. Regardless of this, however, the lack of preliminary data due to the interruption of pilot trials during the pandemic may have long lasting implications for future funding and dissemination of knowledge in this area, ultimately impacting service provision for PwMS.

While we found no gender differences in the number of barriers reported by respondents, our qualitative analysis suggests subtle differences in the types of barriers encountered. For example, only female respondents cited time or competing demands as barriers since COVID-19. We also uncovered some concerning reports of possible gender or career biases that may impede the progression of female and/or early career researchers in the future. While this is partly in keeping with other research which has suggested how the pandemic has disproportionally affected female researchers,^5,6^ we cannot draw firm conclusions regarding gendered effects in this research context specifically. We suggest that this is something worth closely monitoring in the future, particularly regarding whether COVID-19 related interruptions may manifest into lower research outputs for female researchers in the coming years.

### Limitations

Although we contacted many research networks worldwide, we cannot be assured that the sample represents the diversity of MS researchers in the field of rehabilitation and QoL. We also cannot be sure of the response rate to this survey, given the nature of the recruitment strategy employed. Additionally, the cross-sectional design warrants caution when interpreting the retrospective analysis of barriers prior to COVID-19.

### Conclusions

While the existence of barriers to research following COVID-19 echoes findings from other fields,^1,9^ including reports of widespread closure of facilities and limitations accessing funding, we have shown how a number of unique challenges exist for MS rehabilitation/QoL research specifically, including many that existed prior to the pandemic and which therefore may be expected to continue should face-to-face research recommence. Given the call to prioritize areas of research supporting people with progressive MS in particular,^10^ it is vital that any barriers to research in the area of rehabilitation and QoL are tackled. It is also important that such obstacles are taken into account when planning how best to support researchers over the coming years, which will in turn have implications for the successful symptom management and wellbeing of PwMS.

## Supplemental Material

sj-pdf-1-mso-10.1177_20552173211038030 - Supplemental material for Research interrupted: The impact of the COVID-19 pandemic on multiple sclerosis research in the field of rehabilitation and quality of life Click here for additional data file. Supplemental material, sj-pdf-1-mso-10.1177_20552173211038030 for Research interrupted: The impact of the COVID-19 pandemic on multiple sclerosis research in the field of rehabilitation and quality of life by Rebecca Sinead Maguire Valerie J BlKathy Mock Jonsdottir Gloria vonGeldern Michelle Ploughman in Multiple Sclerosis Journal – Experimental, Translational and Clinical
