# Supplementary material for: Research interrupted: The impact of the COVID-19 pandemic on multiple sclerosis research in the field of rehabilitation and quality of life
Source: Mult Scler J Exp Transl Clin. 2021 Aug 26;7(3):20552173211038030. doi: 10.1177/20552173211038030 (PMC8404642; doi:10.1177/20552173211038030)
Supplement: sj-pdf-1-mso-10.1177_20552173211038030 - Supplemental material for Research interrupted: The impact of the COVID-19 pandemic on multiple sclerosis research in the field of rehabilitation and quality of life [file sj-pdf-1-mso-10.1177_20552173211038030.pdf]

Supplementary Appendix – Survey Questions

Demographic and employment-related questions

What is your gender identity?
- Male
- Female
- Non-binary
- Other (specify)

What country/region are you currently working in?
- *Open text response*

What is your career stage?
- Still in training (e.g. PhD or MSc student)
- Early career (5 years or less since first research/academic appointment)
- Mid career (6-12 years since first research/academic appointment)
- Senior researcher (>12 years since first research/academic appointment)
- Other (specify)

What is your current employment status?
- Full-time
- Part-time

What is your contract type?
- Permanent
- Fixed-term
- Other (specify)

Approximately how many years have you been engaged in research (of any type)?
- *Open text response*

Approximately how many years have you been engaged in research specific to rehabilitation/Quality of Life in MS?
- *Open text response*

What percentage of your working time is spent on research activities each week?
- x% time spent on research activities

Do you have any of the following caring responsibilities? Check all that apply
- Children aged 5 years and under
- Children aged 6-12 years
- Children aged 13-17 years
- Older adult care
- Other care (specify)

Barriers to research

Before COVID-19, did you encounter any barriers in conducting research in rehabilitation/QOL in MS?

- Yes, to a great extent
- Yes, to some extent
- No
- Unsure

If yes, outline the main barriers to conducting research before COVID-19.

- *Open text response*

Since COVID-19, did you encounter any barriers in conducting research in rehabilitation/QOL in MS?

- Yes, to a great extent
- Yes, to some extent
- No
- Unsure

If yes, outline the main barriers to conducting research since COVID-19.

- *Open text response*

When compared to researchers of other genders, I have experienced greater difficulties in conducting research in MS rehabilitation following COVID-19.

- Strongly disagree
- Somewhat disagree
- Neither agree nor disagree
- Somewhat agree
- Strongly agree
